# Reshaping Food Policy and Governance to Incentivize and Empower Disadvantaged Groups for Improving Nutrition

**DOI:** 10.3390/nu14030648

**Published:** 2022-02-03

**Authors:** Jingjing Wang, Xinyue Ding, Haixiu Gao, Shenggen Fan

**Affiliations:** Academy of Global Food Economics and Policy, College of Economics and Management, China Agricultural University, Beijing 100083, China; jwang010@cau.edu.cn (J.W.); dxyany@hotmail.com (X.D.); s.fan@cau.edu.cn (S.F.)

**Keywords:** nutrition, food policy, governance, food systems, disadvantaged groups

## Abstract

The coronavirus disease 2019 (COVID-19) pandemic has exacerbated global malnutrition challenges, disrupted food supply chains, and left poor and vulnerable people unable to produce and access safe and affordable food, especially in developing countries. Food policy and governance are currently malfunctioning, despite their recognized roles in improving food security and public nutrition in many local and national contexts. This article reviews existing food policies and governance with implications for disadvantaged groups in the food systems, particularly smallholder farmers, women, and small- and medium-sized enterprises (SMEs), highlighting the importance of reshaping food policies and governance. To end malnutrition in the post-COVID era, multiple sectors, including health, agriculture, social protection, education, and infrastructure, must make greater collaborative efforts to develop and implement food and nutrition policies. Several recommendations for reshaping food policy interventions and governance are summarized.

## 1. Introduction

Nutrition is the key element for the Sustainable Development Goals (SDGs), notably, SDG-2—“End hunger, achieve food security and improved nutrition and promote sustainable agriculture”, and is also essential for the realization of other SDGs [1,2]. Above all, nutrition is the fundamental right of all humankind. It can be viewed as both an input to and an outcome of the SDGs [1,3]. Malnutrition–undernourishment (hunger), micronutrient deficiencies (hidden hunger), and over-nutrition lead to high disease burden and economic costs by adversely affecting individual health, well-being, and productivity [1,4,5,6,7]. In 2020, globally, 149.0 million people were still affected by stunting, 45.4 million (6.7%) were affected by wasting, and more than 340 million suffered from one or more micronutrient deficiencies, including deficiencies in vitamin A, iron, iodine, and zinc [8]. Meanwhile, 2 billion adults worldwide are overweight (39%), and over 670 million are obese (13%) [4]. Overweight, obesity, and other diet-related non-communicable diseases (NCDs) contribute to 4 million deaths annually at the global level [4]. Progress in addressing the problem of malnutrition will have a wide-ranging impact on improving health (SDG-3), improving economic gains, and eradicating poverty (SDG-1).

Factors that contribute to malnutrition are not limited to a lack of food, but also include health care, education, sanitation and hygiene, women’s empowerment, and consumption patterns. Improving nutrition and achieving SDG-2 will depend on progress across other SDGs, including those aimed at clean water and sanitation (SDG-6); renewable energy, education, gender equality and empowerment (SDG-5); and sustainable consumption and production characterized by enhanced food supply chains and safety nets (SDG-12). The co-occurrence of multiple epidemics, including obesity, undernutrition, and climate change, constitutes the global syndemic and affects human and planetary health [9]. The recurrent natural disasters, natural resource degradation, and rapid increase in zoonic diseases constitute a series of epidemics and exert significant pressure on food systems [9,10,11]. Environmental challenges, such as climate change, natural resource depletion, and biodiversity loss, have put future food security and nutrition at greater risk. The most recent report from the Intergovernmental Panel on Climate Change (IPCC) predicts that the climate will change across all regions of the world in the coming decades, and two degrees Celsius of global warming is likely to reach critical thresholds for food and health [12]. The increase in agricultural activities to meet the demands of a growing population, changing diets, lifestyles, and biofuel production puts great pressure on natural resources and ecosystems and further leads to the loss of biodiversity [13,14].

The COVID-19 pandemic further exposed the weakness and fragility of food systems [6,15]. The pandemic disrupted farm production, food processing, transportation, and logistics, leaving poor and vulnerable people unable to produce and access safe and affordable food [16]. The recent increase in unaffordability has aggravated global food insecurity and pushed 83 to 132 million more people into chronic undernourishment [17]. In 2020, 720 to 811 million people worldwide faced hunger, 2.37 billion could not access adequate food [17], and over 3 billion people worldwide could not afford a healthy diet [17,18]. 

Food policy and governance are currently malfunctioning despite their recognized roles in improving food security and public nutrition in many local and national contexts. With malnutrition being a multi-sectoral, multi-stakeholder, and multi-level challenge, policymakers must face trade-offs between personal needs and collective goals, as well as government regulations and voluntary industry codes [10,19]. Many countries have realized that the failures of food policies could distort resource allocation, leading to the overuse of water and land, increased greenhouse gas emissions, and worsening public health [20,21]. Weak governance, rising food prices, and food insecurity may result in marginalization and exclusion [22,23]. Meanwhile, due to their transnational nature, many market and institutional failures cannot be addressed at the national level and thus require global action [24]. One of the major causes of these policy failures is that food and nutrition are related to many “international public goods” (IPGs), such as international trade policies, research and innovation, and transboundary food safety [22,24]. However, without efforts beyond the health sector and joint actions from agriculture, social protection, education, and transportation to implement food and nutrition policies [5,25], the tasks involved in ending malnutrition remain daunting. 

In order to achieve global nutrition targets, food system approaches have been proactively used. The first United Nations Food Systems Summit (UNFSS), held on 23–24 September 2021, suggests maximizing the co-benefits of a food systems approach and transforming food systems to drive our recovery from the pandemic and achieve the SDGs [26,27,28]. With the close participation of all people driving food systems, the process of UNFSS revealed new solutions for multi-stakeholders to work together to further strengthen food systems and support people in their right to food [29]. Today, more than 2 billion people are working in the food sector; 570 million are smallholders, and half of the farmers are women [30]. With the principle of “multi-stakeholder inclusivity”, the UNFSS emphasizes the importance of more equitable progress toward ending malnutrition in all forms [15] and prioritizes the empowerment of essential stakeholders, such as smallholder farmers, women, and vulnerable groups [28,31]. It also highlights the need to tackle emerging nutrition treats using policy interventions to make healthy and less processed food more available, accessible, and affordable [32] and to launch new solutions, actions, and strategies to maximize synergies and minimize trade-offs in the food system [31]. Based on a narrative review, we highlight the critical roles of disadvantaged groups, particularly smallholder farmers, women, and small- and medium-sized enterprises, in food systems to improve nutrition, and discuss potential policies and governance measures to incentivize and empower them to play active roles in nutrition-driven food systems.

The paper contains a state-of-the-art review, a sort of narrative literature review that focuses on recent research and discusses what is currently known and agreed upon for a review topic. We searched Google, Google Scholar, and the official websites of FAO, IFPRI, WHO, UNICEF, and other organizations for studies on “food systems”, “nutrition”, “governance”, “food policy”, and “nutrition-sensitive agriculture”, with a focus on disadvantaged people (smallholder farmers, women, and SMEs). Reports and articles, particularly those published between 2010 and 2021, were chosen when they contained these keywords. Furthermore, for these chosen articles or reports, we swiftly reviewed their references and included some extremely related articles or reports in our reference pool. Finally, this review contains 127 articles or reports.

The remainder of this paper is structured as follows. The following section discusses why food systems are essential for improving nutrition. Section 3 presents how smallholders can be leveraged to improve nutrition. Section 4 highlights the importance of empowering women to improve nutrition. Section 5 reviews strategies incentivizing private sectors, particularly SMEs, to improve nutrition. The last section concludes with a discussion and implications. 

## 2. Reshaping Food Systems to Improve Nutrition

**Figure 1:**
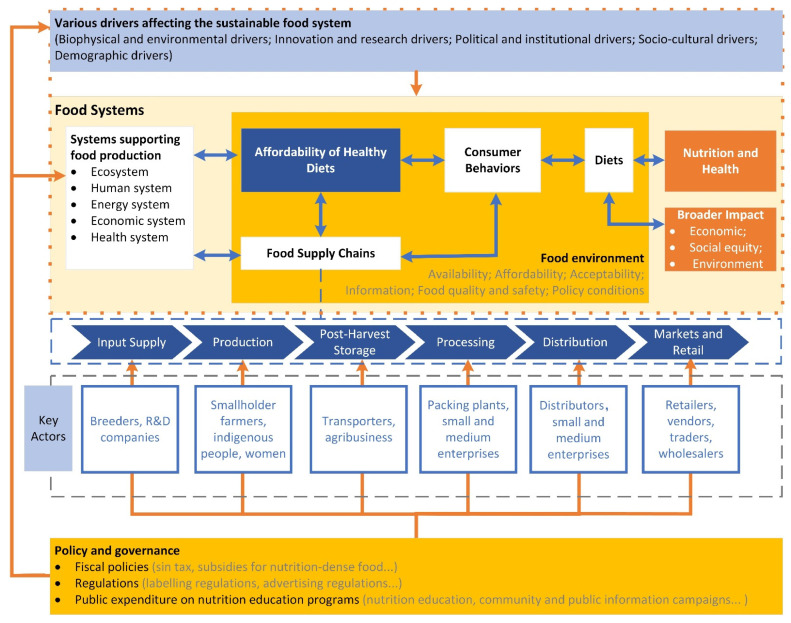
Conceptual framework of food systems to improve nutrition (author’s compilation based on [17,33,34,35,36]).

Food systems include all the actors and activities along global value chains, including input supply, production, processing, distribution, retailing, wholesaling, the preparation of food, and food consumption, together with their impacts on the environment, health, and society [9,27,33]. A sustainable food system aims to ensure the global outcomes of human health, ecological health, social equity, and economic prosperity while minimizing the adverse environmental impact [11]. Figure 1 presents a framework showing how various drivers acting on supply chains, consumer behaviors, and diets can influence food systems and nutrition. 

### 2.1. The Food Supply Chain

The food supply chain, from input supply to production, post-harvest storage to industrial processing, distribution to retail and markets, involves small- to large-scale actors from both the public and private sectors. By increasing access to macro- and micronutrients, food supply chains can improve the nutritional value of food. Meanwhile, the decisions of interacting actors in the food systems influence the food produced or processed in the supply chain and further affect the nutritional value of food [33]. 

Upstream (production)

Agriculture investment and associated research and development (R&D) have a long history of prioritizing foods with potential nutritional values [37]. Even before production, investment in R&D, such as biofortification and cultivar breeding, can provide improved varieties for production. Using more diverse and integrated production strategies, farmers, small- or large-scale, play a significant role in providing nutritious crops by increasing food production diversity. 

Midstream (storage, processing, and distribution)

How food is stored, processed, and distributed also affects food safety, food quality, and the extent of food losses and food waste, and further affects dietary quality [38]. For instance, some perishable foods, such as fruits, vegetables, and animal-sourced foods, are nutrient-dense. However, cold chain storage and transportation are not always available in rural areas, especially in some low- and middle-income countries (LMICs). Food processing can change the nutrient content of food, extend shelf life, and improve palatability and convenience [39,40]. However, it may also decrease the nutritional value by reducing fiber and key nutrients and adding unnecessarily high levels of ingredients that may have health consequences [40]. With the recent development of food value chain approaches, competitive strategies and “upgrading” methods focus on adding value instead of producing healthy, high-quality food [41]. Promisingly, with targeted intervention policies and regulations, private and public actors in the midstream can be essential in providing nutritious food. In particular, storage, processing, and distributing companies (including small- and medium-sized enterprises, packaging plants, and food and beverage companies) can provide nutrition-dense food by food fortification, improving the storage of perishable foods, or reshaping food formulation towards a lower level of substances (e.g., trans fat, and high levels of sodium and sugar) [33].

Downstream (retailing)

Grocery markets and retail outlets are closest to consumers. The retailing sectors can affect food acceptability, consumer preferences, and consumption patterns in various ways. Evidence shows that the ways in which retailers and the market promote food will directly influence children’s preferences, nutrition knowledge, and dietary patterns and further affect their nutrition intake and health [42,43]. Consumers, particularly caregivers, parents, and children, may be drawn to unhealthy food and acquire dietary habits as a result of repeated information created by the advertising, branding, and marketing of unhealthy foods, such as products that are high in fat, sugar, and salt [42,44,45,46]. Small- and medium-sized firms (SMEs) are important linkages between the industry and consumers in developing countries, but they have limited incentives to serve nutritious food to low-income consumers in the absence of targeted food subsidies.

### 2.2. Consumer Behaviors and Diets

Consumers’ food choices and dietary patterns link the food system to individuals’ nutrition and health outcomes. There is consensus that dietary patterns can influence overweight, obesity, micro-nutrient deficiency, and other NCDs. The guidelines from the World Health Organization (WHO) suggest that a healthy diet includes nutrient-rich foods, such as fruits, vegetables, legumes (beans), nuts, and whole grains, with limited intake of sugars, fats, and salt [47]. More recently, dimensions beyond the healthiness of diets have been emphasized; sustainable healthy diets have been promoted to achieve individual health and nutrition and a low environmental impact or stress [48,49]. 

### 2.3. Actors in the Food Systems

Food systems extend far beyond the food value chain, with actors such as producers, distributors, food industry workers, and consumers all playing vital roles. As shown in Figure 1, the food environment, manifested by food acceptability, affordability, information, quality and safety, and policy conditions, is closely connected to food security and nutrition [17]. In the meantime, the food supply chain and the food environment are supported by other systems, including the ecosystem, human system, energy system, economic system, and health system. On the one hand, food systems can have both positive and negative effects on human health via various interconnected pathways; on the other hand, the food system’s operation is dependent on input from healthy and productive individuals. Food system actors from all sectors are working together to make food systems more robust to the key drivers of recent rises in food insecurity and malnutrition, while also enhancing access to affordable healthy diets for all through environmentally sustainable techniques [17].

Among all the participants in the food system, some disadvantaged groups are frequently disregarded and have less opportunity to speak up. The following sections describe how disadvantaged groups, such as smallholder farmers, women, and SMEs, play important roles in food systems, and how food policies and governance can be reshaped to empower and incentivize these groups to improve nutrition for the general population in developing countries. 

Food policies and governance can influence nutritional outcomes by shaping how food systems function and affect other drivers of food systems. In particular, food and agricultural policies, such as food standards, labeling, and reformulation, are important for food availability, food access, and food affordability. More crucially, governance is essential for implementing food system transformations and ensuring that all stakeholders, particularly smallholder farmers, women, and SMEs, can benefit from improved nutrition through shifting the food environment and influencing biophysical and environmental drivers [17]. Therefore, in order to establish an ideal food system delivering high-quality food that is both nutritious and affordable for everyone [6], integrative solutions are needed across the spectrum; multi-sectoral policies including agricultural trade policies, infrastructure policies, and social protection policies are required to improve nutrition [7]. The food systems approach has been proved to be productive and efficient in shifting focus towards nutrition in many countries. For instance, a systems approach that considers policy interventions by different sectors is required to improve the access and affordability of healthy diets more effectively, contributing to a better food security and nutrition situation within the population [50].

## 3. Incentivizing Smallholders to Improve Nutrition

Smallholder farmers (with farmland spanning less than 2 hectares) are critical to eradicating hunger and malnutrition around the world. Even though small farms account for only 12% of the world’s arable land, they generate 30–34% of the world’s food supply, provide livelihoods for over 2 billion people, and produce nearly 80% of the food in Sub-Saharan Africa (SSA) and Asia [51,52]. Many smallholder farmers still struggle to afford enough food and experience food insecurity and malnutrition. A recent study showed that a large proportion of people lacking in certain nutrients (micronutrients) are smallholder farmers in developing countries [53]. Moreover, small-scale farmers face a complex set of risks and challenges that jeopardize their livelihoods, food security, and nutrition [50]. They are increasingly vulnerable to climate change, health challenges, price volatility, and financial instability. Smallholder farmers are the least able to adapt to climate change by investment to continue production [54,55,56]. Studies have found that support and investments in agricultural research, innovative technology, and regulatory reforms are still low in many areas where smallholders predominate [54,57].

During the COVID-19 pandemic, existing constraints faced by smallholders have been exacerbated [9]. Therefore, strengthening smallholder agriculture is essential to ensuring food security and nutrition and improving rural livelihoods [58]. Most existing agricultural subsidy policies focus on staple grains in many countries without considering vegetables, fruits and beans, and other foods with high nutritional value. As a result, one of the most pressing issues is making smallholder agriculture more nutritionally sensitive [41]. We summarize some policies or governance to help make smallholder agriculture more nutritionally sensitive. These policies or governance would affect smallholder farmers’ food production behavior, labor allocation within households, and agricultural income, which will directly or indirectly contribute to nutritional outcomes [59].

### 3.1. Greater Investment in Rural Infrastructure to Connect Farmers to Food Markets

Inadequate rural infrastructure leaves communities isolated, holds back food value-chain development, contributes to post-harvest food losses, and is significantly associated with poverty and poor nutrition [23,60]. Investment in rural roads allows perishable nutritious foods, such as vegetables and fruits, to be delivered to towns and counties promptly [23]. Cold storage infrastructure can prolong the preservation time of agricultural products, ensure the nutrition of products, give farmers new marketing options, and help farmers choose the right time to sell at a better price and increase their income [61]. Government investments should not only include traditional physical infrastructure, but also contain information and communication infrastructure to leverage the potential of digital technology. Information and communications technology (ICT) connectivity allows farmers to access many other valuable sources [23]. Nigeria, for example, has introduced an electronic wallet program that delivers seed and fertilizer vouchers directly to farmers’ mobile phones, and it has been extended to include additional benefits, such as dietary supplement vouchers [62].

### 3.2. Support for Innovative Technologies to Improve Nutrition 

Evidence shows that innovative technologies can improve food safety and nutrition [63,64,65]. For example, biofortification shows particular promise for bringing smallholders into “healthy” value chains that promote a nutritious diet, from seeds to consumption [23,66]. Biofortification strategies can be achieved through agronomic practices, breeding, and genetic biotechnology approaches, and each has its advantages and disadvantages [67,68]. Biofortification has proven helpful in addressing hidden hunger [68]. Recent research found that smallholder farmers adopting and producing biofortified crops in Uganda can prevent child stunting [69]. The limitations of biofortification include micronutrient stability and bioavailability in crops, as well as farmers’ adoption and acceptability of modified crops, and the public who worry about their safety [67,68,70]. Concerns about genetically modified (GM) crops include transgenic effects on the natural landscape, the significance of gene flow, biodiversity consequences, and farmers’ reliance on GM seeds of a few large firms [71,72]. GM crops may pose a significant danger to the global economy [72]. Therefore, countries should establish effective and transparent regulatory and monitoring systems to govern emerging technologies. Thus far, GM crops that have been commercialized have been deregulated and certified safe for the environment and human health by competent authorities worldwide, including the European Food Safety Association [72], while regulators must continue to exercise vigilance to ensure that no GM crops that may threaten human health or the environment are deregulated [72,73]. 

### 3.3. Promoting Production Diversity and Market Access to Increase Smallholder Consumption of Nutritious Foods

More diverse autochthonous local crops and animal breeds can increase the resilience of food systems, and their inclusion in diets could reduce nutrient deficiencies [74,75]. Promoting nutrient-rich crops through home gardens and diversifying the production system could enhance nutrition [76]. Given that smallholder farmers typically consume part of what they produce at home, increasing production diversity on their farms is often seen as an effective solution to improve smallholder nutritional outcomes [77,78]. The situation is different when one considers the impact of market access on the nutritional status of smallholder farmers. The latest research shows that the effectiveness of increased production diversity reflected in improved home nutrition depends on market participation and market access [79,80]. An important policy implication is that improving market access is key to making smallholder agriculture, especially for subsistence farms, more nutritionally sensitive [80]. In order to facilitate market access and income for smallholders, rural infrastructure should be designed to be consistent with support measures to provide households with affordable and nutritious food. Helping farmers to meet higher food quality standards through regulation and quality certification can also support market access [23]. 

### 3.4. Commercialization and Complementary Actions Help Smallholder Production to Achieve Nutritional Transformation

Commercialization in the small farm sector improves dietary quality by increasing calorie, zinc, and iron consumption [53]. However, commercialization alone is insufficient to address all types of malnutrition; complementary interventions may also be needed [53]. Contract farming, agricultural extension systems, and primary health care are proven to improve smallholder nutrition. Debela et al. found that contract farming improves household- and individual-level diets and nutrition; the effects vary depending on the contract type [81]. Redesigning agricultural extension systems to increase nutrition promotion knowledge might help to improve the nutrition of smallholder farmers. Investments in primary health care, clean water and sanitation, childcare, and hygiene can also help to make smallholder farming more nutritionally sensitive [66].

Previous studies have shown that agriculture-nutrition policy and governance, which improved smallholders’ agriculture productivity and market access, greatly impacted food security and nutritional outcomes. However, no single intervention can address all the complex challenges smallholder farmers face. Several aspects need to be considered, researched, intervened in, or governed in the future to enable smallholder farmers to counter the threat of COVID-19 to rural livelihoods and achieve food security and nutrition. First, it is insufficient to develop new technologies; instead, the government should ensure that they are available to smallholders [82]. Second, long-term investment for structural changes and public investment in health services are needed to improve smallholders’ productivity and the well-being of other less powerful actors along with the food system [83]. Third, the effects of institutional innovations combined with digital technology, such as expanding the content of agriculture extension through ICTs and conducting nutrition education or training, on smallholder farmers’ income and nutritional outcomes ought to be studied by researchers.

## 4. Empowering Women to Improve Nutrition

Gender equality and empowerment are critical contributors to good nutrition [84]. Empowering women is crucial to improve nutrition for families and societies. On the one hand, women are vulnerable groups as they have higher nutritional needs due to physiological requirements, particularly during pregnancy and breastfeeding. On the other hand, empowering women can improve nutrition for other household members, especially their children, as women are often their families’ primary caregivers and food providers. Women play a vital role in children’s growth and health. When women suffer from undernutrition, their offspring are more likely to suffer from malnutrition in their first 1000 days post-conception [85]. Furthermore, the critical window of “1000 days” regarding developmental damage resulting from undernutrition cannot be reversed and will affect their quality of life for a lifetime [86,87,88]. Empirical evidence shows that empowering women should be the core of all efforts to improve nutrition for mothers, their children, and other household members by strengthening women’s ability to provide food security, health, and nutrition for their families [89,90]. More than half of the reduction in child stunting from 1970 to 1995 can be attributed to increases in women’s status [91]. Research further found that empowering women to achieve gender equality would reduce child malnutrition by 13% in South Asia and by 3% in Sub-Saharan Africa [92]. Additionally, as producers, women play a vital role in improving agricultural output and food supply diversity. In developing countries, smallholder women make up 43% of the agricultural workforce. However, women usually have lower food productivity and food availability, which can be improved by improving their production skills and increasing land ownership. Increased food productivity and food availability among women can further improve their nutrition and health and that of their families [93]. When women are producers, their impact on nutrition and health is via increased food access, which is similar to that of smallholders, so this is not emphasized in this section.

**Figure 2:**
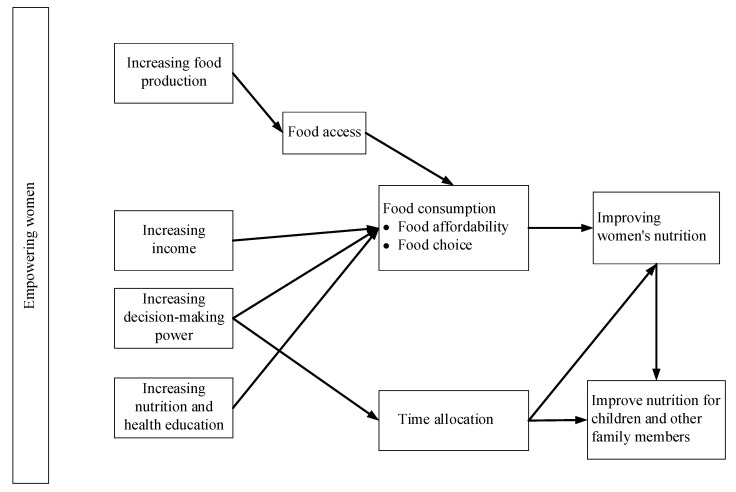
Empowering women to improve nutrition (author’s compilation based on [96,97,98,99]).

Despite women’s vital role in agricultural production and household food and nutrition security, these contributions are often not formally recognized [23,94]. In many countries, women have lower education levels than men; have fewer resources to control; and have less decision-making power over household income and time [23]. This puts women at a higher risk of malnutrition, as their physiological needs are different from those of men, and they are frequently misunderstood and underserved [88], which further increases the risk of child malnutrition and deaths [95]. Therefore, many studies and programs have focused on women’s empowerment to address gender inequalities and improve nutrition and health. The results suggest that (a) empowering women can increase their incomes, as well as their decision-making power in their households and communities, stimulating a virtuous cycle of economic empowerment; (b) even if their income increases, whether a woman will use it to improve her or her family’s nutritional status depends mainly on her health awareness [23,90]. In Figure 2, we summarize the main pathways to improve women’s and their children’s nutrition and health by empowering women. Additionally, here, we focus on explaining the effects of women as consumers, namely increasing income, nutrition and health education, and decision-making power.

First, regarding increasing women’s income, studies have found that when women’s income increased, their nutritional status and that of their family increased. Hallman found that a higher share of women’s assets is associated with better health outcomes for children [100]. Moreover, homestead food production projects in Bangladesh have resulted in a higher vitamin A status and energy intakes for vulnerable household members by increasing women’s income [90]. Furthermore, compared to men, women tend to spend their additional income on food and health care and improved nutritional outcomes in the household [92].

Second, improving girls’ and women’s access to education significantly increases their nutrition and health education. Studies have found that, even if women’s incomes increase, they still might lack the health awareness to actively improve their nutrition status [101]. Therefore, increasing women’s nutrition and health education is a crucial pathway to improving nutrition by empowering women. For instance, through increased year-round food availability and nutrition education, homestead food production projects in Bangladesh helped to ensure that nutritious foods were eaten instead of sold to achieve positive nutritional outcomes [90].

Third, it is critical to increase women’s decision-making power, especially in the family. Studies have found that empowering women in purchasing decisions, health care decisions, family planning decisions, and spousal communication can improve their nutrition and health and that of their children [102,103,104,105,106,107]. For example, the Enhanced Homestead Food Production (E-HFP) program in Burkina Faso empowers women by (1) increasing agricultural training and inputs for women (e.g., tools, seeds, and chickens) to promote the production of nutrient-rich foods (e.g., eggs and vegetables) for sale and consumption; (2) behavior change communication planning to foster the adoption of optimal health and nutrition practices; (3) developing land-use agreements to facilitate women’s access to agricultural land, and found that women’s empowerment is a pathway by which a nutrition-sensitive program can avoid child wasting [108]. 

Moreover, intra-household time allocation can affect women’s and their families’ nutritional outcomes. For example, encouraging men to increase their contribution to family work effectively improves the nutritional status of family members when women spend less time caring for the home. A large amount of research has shown that increased maternal working time affects the risk of childhood overweight and obesity [109,110]. However, the correlation was not significant in a Danish study; the main reason for this is that Danish fathers contribute significantly to their children’s health [111]. The COVID-19 pandemic has added to the burden of unpaid domestic and care work, and women already spend about 2.5 times as many hours on this as men [112]. Therefore, policies focusing on reducing the working time of breastfeeding mothers and encouraging men to take on increased domestic work and childcare should be considered.

Despite the large amount of research on agricultural and infrastructure development focusing on gender equality, we found that many programs and policies to improve gender equity are “unfinished business”, or the results of interventions have not been reported [90]. Some programs and policies have found that the expected results have not been achieved, even though they have increased women’s incomes and nutrition knowledge. The reason for this is that there are still other necessities in the family that have not been solved or improved. For example, the U.S. Agency for International Development (USAID) Family Farming Project in Tajikistan between 2010 and 2014—implemented by DAI, Winrock International, and Save the Children International—provided leadership opportunities for women and improved their status in their communities and families. The interventions included training on crop production, savings groups, infant and young child feeding, and food preservation training. Although these projects increased women’s nutrition knowledge, the goal of improving nutrition was not achieved because women still had a more urgent life need—purchasing safe drinking water—that prevented them from fully utilizing their knowledge of nutritional practices [90].

To summarize, many interventions or programs focus on improving nutrition by empowering women, but challenges remain. Based on past intervention studies and programs on intervention outcomes, suggested directions for future intervening measures include: (a) increasing women’s income, (b) increasing women’s nutrition and health education, and (c) increasing women’s decision-making power in the family. The implementation of the above measures requires the joint support of policy and the social environment, which means that we need to increase our attention on improving gender policies and empowerment. Additionally, we should attach great importance to tracking the results of interventions to summarize experiences. 

## 5. Enabling Small- and Medium-Sized Enterprises to Improve Nutrition

Small- and medium-sized enterprises (SMEs) are on the frontline of tackling malnutrition, especially in emerging economies [113,114]. Agri-food SMEs, in farming, transportation, processing, and distribution, can be essential for well-functioning food systems as well as food and nutrition security [115]. They can also promote the inclusion of the rural poor population by expanding the “hidden middle” of the supply chain [23]. Since food processing, distribution, and services are labor-intensive, these industries can create off-farm employment opportunities in rural and suburban areas. They act as virtual nodes in a network of small- to medium-scale producers, processors, aggregators, distributors, and retailers that collectively manage the flow of nutritious foods from farm to plate [116]. In LMICs, as much as 80% of all food consumed is handled by SMEs [113], and 50–60% of the workforce in Sub-Saharan Africa and Southeast Asia are employed in SMEs [117]. Thus, the failure of these SMEs would jeopardize food and nutrition security for millions of people [118]. More recently, SMEs have provided various forms of agricultural services, including seeding, spraying, pruning, land preparation, harvesting, and marketing, which are traditionally carried out by farmers themselves [119]. Compared to large enterprises offering a variety of services, SMEs in developing countries can benefit disadvantaged groups, including farmers, ethnic minorities, and women, by providing tailored services with improved access [120].

**Figure 3:**
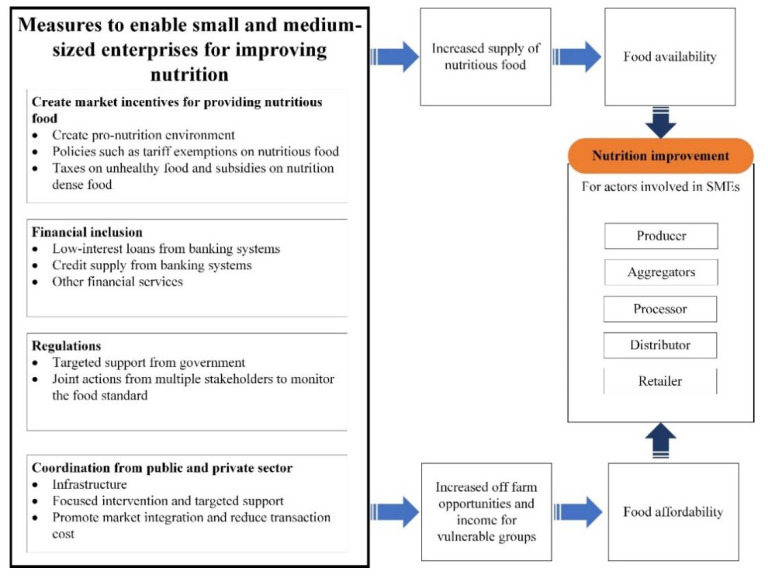
Enabling small- and medium-sized enterprises to improve nutrition.

Nevertheless, the supply chain dominated by SMEs is vulnerable and is often neglected. With infrastructure limitations, these systems are poorly integrated, heavily dependent on hired labor, and more vulnerable to disruptions in input supply [41]. The COVID-19 pandemic introduced an acute shock to businesses worldwide, revealing multiple challenges for SMEs. As one of the important investors in the food system and in line with the goal of maximizing profit, SMEs should be equipped, beyond basic training, to create lasting impacts and inspiration to supply nutritious and safe foods. Existing studies suggest that the following policies and governance can help to improve the food environment and the supply chains through SMEs (as shown in Figure 3). 

### 5.1. Create Market Incentives to Provide Nutritious Foods

Incentives will be the key in encouraging businesses to reshape their goal toward nutrition sensitivity [23,121]. For a long time, the food sectors in many developing countries have focused on supplying enough staple food. More recently, businesses along the food value chain have often been driven by profits and have found no incentives to identify the added value of nutritious food. Most of them mainly focus on producing and distributing highly processed food [36,46], as these foods are easy to transport and produce at a large scale [121]. 

With the aim to build a nutritious food system, private businesses, including SMEs, should reshape their priorities toward producing nutrient-dense food. The business environment can be reoriented to foster actions supporting nutrition improvement [34]. In LMICs, SMEs’ choices, such as marketing ultra-processed food with loaded sugar, salt, and fat to children or providing nutritious and healthy foods such as fruits, vegetables, beans, and whole grains, will lead to dramatically different nutritional outcomes [34]. One way to incentivize the provision of nutrient-dense food from the private sector is to create market demand for nutritious food [121]. 

To further encourage SMEs to provide more nutritious food, the government can use fiscal policies, contribute to the development of food value and cultures, and organize consumers and investors to reward or penalize SMEs [34]. For instance, many countries have already applied taxes on unhealthy food and drinks, such as “sugar taxes”. Researchers are also modeling the effects of adopting healthy food subsidies on fruits and vegetables [122]. Although the related health improvements have not been fully established, these policies greatly reduce the consumption of sugar-sweetened beverages (SSBs) and other products with high levels of added sugar [123]. With the industry levy and decreasing demand for unhealthy food, the food industry, including SMEs, will be encouraged to work on the reformulation of food with less fat/salt/sugar and more fiber and micronutrients [34]. Therefore, governments must emphasize the importance of nutrition when creating policies and apply a food-based dietary guidelines lens to fiscal policies [29]. The collected tax revenues can be used to promote the production and retailing of healthier food products, e.g., whole grains, fruits, and vegetables, and to promote industry reformulation.

### 5.2. Provide Infrastructure, Financial, and Technical Support for SMEs 

In many developing countries, SMEs face the obstacles of unfinished infrastructure and limited access to the market. Most small- and medium-sized businesses find it hard to access financial services [121]. Due to their small size and the lack of essential technical skills, SMEs are often unable to bear such risks. As the pandemic has impacted their operations, the lack of operational cash flow reduced SMEs’ resilience and ability to tackle the crisis [124]. Therefore, support from multilateral institutions, especially governments and banking systems, is needed for SMEs to overcome the obstacles to access credit, capital, and insurance. Meanwhile, the susceptibility of SMEs’ major clients, smallholder farmers, and people in rural areas to shocks and crisis further threaten SMEs’ ability to raise revenue and continue operating, and thus affect the ability of consumers to access nutritious foods [116]. In these cases, interventions to bolster consumer demand—including unemployment insurance and other social safety nets—can thus be crucial to protecting the most vulnerable populations and also lead to gains in welfare and food and nutrition security [115]. 

With access to improved infrastructure and credits, SMEs can thrive and become instrumental in food provision. Short food supply chains (SFSCs) enable SMEs to retain a higher proportion of added value while connecting farmers and final consumers more directly [125]. Since 2013, the Global Alliance for Improved Nutrition (GAIN) has been working with private firms to serve more nutritious and affordable food in five countries in Africa and Asia [121]. During the pandemic, many countries have provided SMEs with fiscal support, such as providing stimulus packages, easing or delaying loan payments, and providing cash incentives [23]. GAIN provides SMEs with direct financial support, technical assistance, and knowledge centers to support the SMEs in enduring the COVID-19 crisis and building back stronger [117]. The financial and technical support from the public and private sectors can help SMEs produce more available, affordable, desirable, and profitable food [121]. 

### 5.3. Propose Strict Regulations

Government regulations and oversights are critical in delivering safe and nutritious food [41]. As the primary food provider in rural areas in most developing countries, SMEs must follow the necessary food safety and quality standards and market regulations. However, appropriate standards and regulations should be established for marketing, labeling, fortification, and additives, including trans fat, sodium, and added sugars [34]. However, as SMEs may not always have the competence to implement regulations, some unnecessary regulations and informal restrictions might stifle SMEs’ growth.

Therefore, governments should act as facilitators to provide targeted support and regulations to increase the capacity of SMEs. Multiple stakeholders, including non-governmental organizations (NGOs) and civil societies, should also monitor food and ensure that the regulations are both required and valuable in shaping food production toward nutrition. The mandatory labeling of trans fats, for example, has been shown to be successful in reducing trans fat availability in the food supply and influencing industry behavior by driving product reformulation [42].

### 5.4. Foster Public-Private Engagement

More collaborations between actors working on nutrition in public and private sectors are needed to improve nutrition through SMEs [121]. Currently, the food supply chain is still dominated by multinational companies and supermarkets. The public and private sectors may have major differences in culture, language, and networks, making it difficult for SMEs to get involved and determine which foods to produce. Dialogues, including conference panels and a joint proposal from the public and private sectors, are beneficial in providing opportunities for SMEs to communicate and speak out. In 2021, UNFSS called for actions from multi-stakeholders and more than 600 independent dialogues were organized before the FSS. Meanwhile, public–private partnerships can facilitate the construction of the building of roads, ports, and other infrastructure and support the involvement of SMEs in the food systems. For instance, with joint work from civil society organizations, researchers, technologists, academia, government, development patterners, and investors, the Scaling Up Nutrition (SUN) Business Network (SBN) has collaborated to support nutrition-sensitive SMEs in Tanzania in scaling their impact [126]. Public information campaigns will also motivate technological innovation. In addition to promoting links between private actors along the supply chain, stakeholders in food systems should facilitate partnerships between private and public bodies, development agencies, and civil society organizations to push forward critical advances in technology, productivity, and other outcomes. 

In sum, SMEs play an essential role in providing nonfarm employment opportunities, linking smallholder farmers to the market, and reorienting the local food system toward nutrition. During the COVID-19 pandemic, some SMEs bounced back quickly, with the help of innovative technologies, such as e-commerce and ICTs, to adjust their operations and keep their business running. Future policies should address the needs of private stakeholders and focus on providing market incentives, financial and technical support, and food safety standards and regulations to rebuild food systems after the crisis. Well-functioning markets and partnerships must support global, national, and local food supply chains and an environment that allows food system entrepreneurs to promote long-term, market-based solutions [61].

## 6. Discussion and Conclusions

Food systems are facing emerging risks, including climate change, natural resource depletion, undernourishment, malnutrition, and biodiversity loss. To improve food security, food safety, and nutrition, immediate action from multiple stakeholders and government policies are required [127]. Food policy interventions, including those in fiscal policies, research and innovation, investment and financial support, empowerment, nutrition education, and regulation, often have limited effects on the marginalized groups we emphasized in this study. The last three sections conclude with highlights of future food policy and management interventions or research subjects. Reshaping policies and governance can be vital in improving the nutritional outcomes of these disadvantaged groups, which in turn affects the nutritional and health conditions of other important actors in the food systems.

In order to strengthen the nutrition sensitivity of food systems and to achieve the SDG goals by 2030, local, national, and global public policies should be reshaped towards achieving better nutrition. Several recommendations to reshape policy and governance are summarized in Table 1: (a) repurposing agricultural subsidies and reallocating the tax revenues from the food industry to promote the production and industry reformulation of healthy food products; (b) increasing the application of innovative technologies to improve nutrition, such as biofortification; (c) increasing investment in infrastructure, communication technology systems, and financial support; (d) empowering marginalized groups through education, policy, and initiatives; and (e) establishing appropriate industry standards for fortification, labeling, and marketing. Governments and civil societies should reshape policy implementations based on a better understanding of incentives and the interests of actors in the food system. Multiple measures can be taken to reinforce nutrition governance, such as establishing more broad-based dialogues, creating institutional coordination beyond the health sector, and redesigning the governance based on legitimacy, accountability, effectiveness, and inventiveness.

## Figures and Tables

**Table 1:** Reshaping food policy and governance to empower smallholder farmers, women, and small- and medium-sized enterprises to improve nutrition.

Policy Strategies for Different Groups	Failures of Existing Policy and Governance	Reshaping Policy and Governance
Fiscal incentives and disincentives		
Smallholder farmers	Subsidy policies only focused on staple grains; other foods of higher nutritional value are not included.	Increase subsidies for vegetables, fruits, beans, and other nutritious products.
Women	--	--
SMEs	(a) The industry levy on unhealthy food hardly shifted the focus of the food environment toward nutrition; such taxes are generally regressive, putting more financial burdens on lower-income individuals. (b) SMEs have few incentives to provide nutritious food.	Tax revenues can be used to promote the production and retailing of healthier food products, e.g., whole grains, fruits, and vegetables, and to promote industry reformulation.
Research and innovation		
Smallholder farmers	Lack of targeted policies to help smallholder farmers apply techniques.	Increase agricultural R&D, application, and extension of innovative nutritional improvement technologies.
Women	--	--
SMEs	SMEs lack the capacity to apply research and innovation to preserve food structure, aromas, and food nutrients during food processing.	Innovative strategies can be applied to improve nutrition (e.g., sustainable food processing and efficient post-harvest handling by improving cold chain storage and distribution conditions).
Investment and financial support		
Smallholder farmers	Insufficient investment in infrastructure to support the development of nutritionally sensitive agriculture.	Increase investment in rural infrastructure, physical facilities, and information and communication technology systems.
Women	Lack of targeted support for women.	(a) Increase investment and financial support targeted to women. (b) Increase the cooperation of multiple sectors focusing on women.
SMEs	There is insufficient support in credit, capital, and insurance for SMEs to bear the risks.	(c) Increase investment in infrastructure and targeted financial support to SMEs from government and banking systems (e.g., more investment in cold chain storage facilities).
Empowerment and education		
Smallholder farmers	Lack of empowerment policies specifically for female farmers.	Emphasis should be placed on the role of women in agricultural production and family nutrition.
Women	(a) The results have not been reported. (b) Lack of supplementary guidance for health education.	(a) Add relevant research. (b) Increase supplementary guidance for health education.
SMEs	--	--
Regulation		
Smallholder farmers	--	--
Women	--	--
SMEs	(a) Lack of targeted standards or regulations for SMEs. (b) Some SMEs lack the capacity to implement regulations when support, law enforcement, and accountability do not exist.	(a) Appropriate standards and regulations should be established for marketing, labeling, and fortification, and for additives including trans fat, sodium, and added sugars. (b) The capacity of SMEs should be improved through government support.

## Data Availability

Not applicable.
